# Advanced cloud intrusion detection framework using graph based features transformers and contrastive learning

**DOI:** 10.1038/s41598-025-07956-w

**Published:** 2025-07-01

**Authors:** Vijay Govindarajan, Junaid Hussain Muzamal

**Affiliations:** 1 Colorado State University, Seattle, USA; 2 National University of Computer and Emerging Sciences, Lahore, Pakistan

**Keywords:** Computer science, Information technology

## Abstract

This paper presents a modular and scalable intrusion detection framework that combines graph-based feature extraction, Transformer-based autoencoding, and contrastive learning to improve detection accuracy in cloud environments. Network flows are modeled as graphs to capture relational patterns among IP addresses and services, and a Graph Neural Network (GNN) is used to extract structured embeddings. These embeddings are refined through a Transformer-based autoencoder to preserve contextual information, while contrastive learning enforces clear class separation during classification. The system is evaluated on NSL-KDD and CIC-IDS2018 datasets under both binary and multi-class scenarios. Experimental results show an average accuracy of 99.97%, with high precision and recall across all attack types, including minority classes such as U2R and R2L. The model achieves low false-positive rates and demonstrates real-time inference performance with modest resource requirements. Key contributions include an interpretable pipeline using SHAP for feature attribution, a strategy for mitigating class imbalance, and validation across datasets with detailed security and generalizability analyses. These results support the practical applicability of the proposed approach in high-throughput, cloud-based network environments.

## Introduction

The rapid growth of cloud computing and its adoption in various industries have led to significant advancements in scalability, cost-efficiency, and accessibility^1^. However, this widespread reliance on cloud environments also increases the attack surface for cyber threats, making intrusion detection an ever more important component of cloud security. Despite the proliferation of security measures, the fast pace and distributed nature of cloud infrastructure introduces unique challenges. Traditional intrusion detection systems, designed for static networks, struggle to keep pace with the complex patterns of modern attacks^2^. Attackers are employing increasingly sophisticated tactics, ranging from zero-day vulnerabilities to advanced persistent threats (APTs), often targeting the inherent trust within cloud tenants and service providers^3^. This evolving threat field is further complicated by the sheer volume of network traffic and the diversity of protocols and services found in cloud ecosystems. As a result, the ability to detect and respond to malicious activities in real time is important^4^. The limitations of existing methods show the need for innovative approaches that can handle large-scale data, adapt to arising attack patterns, and provide actionable insights for security administrators^5^.

In this context, the use of advanced machine learning techniques and state-of-the-art neural architectures offers a promising path forward^6^. Graph-based representations of network traffic and deep learning models have shown potential in extracting meaningful patterns and identifying anomalies that traditional rule-based systems might miss^7^. By integrating these modern computational methods into the cloud intrusion detection framework, we can begin to address the shortcomings of conventional solutions and move closer to a more secure cloud environment^8^.

### Research problem

Given the increased reliance on cloud infrastructure, the detection and mitigation of malicious activities within such environments remain significant challenges^9^. The key problem lies in developing a robust, scalable and adaptive intrusion detection mechanism that can accurately classify normal and attack traffic in real time, while also providing high precision and recall^10^. Existing systems struggle with handling large datasets, differentiating between benign anomalies and actual attacks, and adapting to the evolving tactics of adversaries^11^. As a result, there is a pressing need for an intrusion detection approach that not only achieves high detection accuracy but also maintains efficiency and interpretability in complex cloud-based scenarios.

### Related work and gaps

Existing solutions to intrusion detection in cloud environments primarily involve signature-based methods, rule-based systems, or traditional statistical models^12^. These approaches often rely on predefined attack signatures or simplistic anomaly thresholds, which limits their ability to detect novel or evolving threats^13^. Although recent advances in machine learning, such as supervised learning models and deep neural networks, have improved detection rates, they frequently suffer from high false-positive rates, lack interpretability, and struggle with generalizing to unseen attack types^14^. Furthermore, many current methods fail to incorporate the rich relational information embedded in network traffic data, which could otherwise enhance their understanding of attack patterns and reduce misclassification^15^.

### Proposed approach

To address these limitations, we propose a novel cloud intrusion detection framework that uses graph-based feature extraction, transformer-based autoencoders, and contrastive learning to deliver a more robust and adaptive solution. By representing network traffic as graphs, our approach captures the relational structure among nodes, enabling more accurate detection of complex attack patterns. The use of transformer architectures allows for refined feature representations that retain important contextual information, while contrastive learning enhances the model’s ability to distinguish between subtle differences in traffic behaviors. This holistic approach not only improves detection accuracy and precision but also reduces false positives, enhances scalability, and provides greater resilience to evolving attack techniques.

### Aim

The aim of this research is to develop a robust, scalable, and interpretable intrusion detection framework that improves detection accuracy and reduces false positives in cloud environments.

### Research objectives

1. To design a graph-based feature extraction pipeline that captures the relational structure of network traffic in cloud environments.
2. To develop a Transformer-based autoencoder for refining feature representations and enhancing model robustness.
3. To integrate contrastive learning techniques into the detection framework to improve classification performance and resilience to evolving attack patterns.

### Research questions

1. How can graph-based representations of network traffic improve the accuracy and interpretability of intrusion detection in cloud environments?
2. What role do Transformer-based architectures play in refining features and reducing false positives in the detection pipeline?
3. How does the integration of contrastive learning affect the model’s ability to adapt to arising attack strategies?

### Significance of research

This research contributes significantly to the field of cloud security by addressing important gaps in current intrusion detection methodologies. By using graph-based feature extraction and Transformer architectures, the proposed framework offers a novel way to understand and detect complex attack patterns. This approach not only improves detection accuracy but also enhances the scalability and adaptability of intrusion detection systems, ensuring they remain effective as cloud environments continue to evolve. Furthermore, the integration of contrastive learning introduces a more refined method for distinguishing malicious traffic from benign anomalies, reducing false positives and enabling security administrators to focus their efforts on true threats. The proposed framework thus provides a more reliable and actionable solution for maintaining trust and security in cloud infrastructures.

The outcomes of this research extend beyond academic contributions, offering practical tools and techniques that can be adopted by industry practitioners. By enhancing the precision, recall, and interpretability of intrusion detection systems, this work helps organizations protect their sensitive data, maintain service availability, and prevent costly breaches. In doing so, it advances the state of the art in cloud security and sets the stage for future innovations in this important domain. The rest of this paper is structured as follows. Section “Background and motivation” provides a detailed review of related work, highlighting existing solutions and identifying their limitations. Section “Research problem” describes the proposed methodology, including the overall framework, data pre-processing, and model components. Section “Related work and gaps” outlines the experimental setup, including datasets, training procedures, and evaluation metrics. Section “Proposed approach” presents the results and discusses the key findings. Finally, section “Aim” concludes the paper and propose directions for future research. To ensure the practical utility and transparency of our approach, the paper further discusses the interpretability of model outputs, evaluates generalization across datasets, and analyzes computational trade-offs. Formal and informal security analyses are included to assess system robustness, and overfitting mitigation strategies are detailed. Limitations related to detecting advanced threats such as APTs are acknowledged, along with directions for future research. Together, these additions aim to present a comprehensive, explainable, and operationally feasible solution for modern intrusion detection.

## Literature review

The advent of deep learning has significantly influenced the field of intrusion detection, offering innovative approaches to identifying and mitigating threats within complex network environments. Generally, deep learning-based methodologies can be divided into two broad categories: supervised and unsupervised techniques. The fundamental distinction between these approaches lies in the availability of labeled data during training. In supervised learning, labeled data guide the model to learn a mapping from inputs to their corresponding output classes. Convolutional neural networks (CNNs), known for their hierarchical feature extraction capabilities, fall into this category and have been widely employed for image-related tasks, but have also been adapted for certain intrusion detection scenarios. Unsupervised methods operate without labeled data, often relying on structures such as deep belief networks (DBNs), autoencoders (AEs), and recurrent neural networks (RNNs). These unsupervised approaches are particularly valuable for uncovering hidden patterns, reducing dimensionality, and detecting anomalies without requiring a predefined ground truth.

Several studies have specifically focused on intrusion detection using benchmark datasets such as KDD Cup 99 and NSL-KDD, which provide well-defined feature sets and labels. Researchers have explored various deep learning architectures on these datasets, demonstrating the versatility and effectiveness of different model configurations. For example, Tang et al.^16^ used a deep neural network (DNN) model on the NSL-KDD dataset to perform anomaly detection in software-defined networking (SDN) environments, selecting six features from the original 41. Although their work confirmed the potential of DNNs for anomaly detection, they encountered high false positive rates when the model was applied outside its trained environment, and the limited set of features posed challenges in identifying more complex attacks. Similarly, Salama et al.^17^ proposed a hybrid approach that integrated DBNs for feature extraction with SVMs for classification. This method improved detection performance compared to using DBNs or SVMs independently, but the combination of multiple approaches added complexity and raised concerns about maintaining real-time performance.

Aygun et al.^18^ introduced autoencoder-based methods to detect zero-day attacks, including standard autoencoders and denoising variants, achieving classification accuracies of 88.28% and 88.65% on the NSL-KDD dataset. Using a stochastic thresholding approach, they improved the models’ ability to handle anomalies; however, this technique did not generalize well across other data sets or real-world conditions. Javaid et al.^19^ utilized self-taught learning (STL) with sparse autoencoders and softmax regression, yielding strong classification results on multiple class categories. Despite achieving satisfactory accuracy, precision, recall, and F1-scores, their method struggled with false positives and difficulties in selecting features for diverse attack scenarios. In another study, Niyaz et al.^20^ applied stacked autoencoders to detect DDoS attacks within an SDN environment. Although the model achieved high accuracy with low false positives, it relied heavily on high-quality labeled data, and its effectiveness varied depending on attack type and context. Wenjuan et al.^21^ introduce a hybrid intrusion detection model using Stacked Contractive Auto-Encoders (SCAE) for feature extraction and SVM for classification. The model shows high performance on NSL-KDD and KDD Cup 99 datasets, achieving excellent AUC scores in 5-class and 13-class tasks, with strong accuracy and recall for DOS and Probe attacks. It also excels in 2-class and 5-class tasks on KDD Cup 99, highlighting its capability with high-dimensional data. Limitations include challenges in detecting underrepresented attack classes (U2R, R2L) and high computational costs for deeper architectures.

Beyond the NSL-KDD dataset, other works have investigated intrusion detection using the KDD Cup 99 dataset. Kim et al.^22^ focused on (APTs) and proposed a DNN model that employed 100 hidden units, the rectified linear unit (ReLU) activation function, and the ADAM optimizer. Although their approach was implemented on a GPU using TensorFlow, improving detection accuracy, it faced high false alarm rates in practical scenarios. Papamartzivanos et al.^23^ merged the KDD Cup 99 and NSL-KDD datasets to create a larger training corpus, enabling them to develop a scalable intrusion detection system based on sparse autoencoders and the MAPE-K framework. Despite achieving an adaptive detection rate of 73.37%, their method required significant computational resources and struggled in highly fast-paced environments. Shone et al.^24^ introduced a non-symmetric deep autoencoder (NDAE) that provided efficient dimensionality reduction and outperformed traditional autoencoders when paired with Random Forest classifiers. Although this approach demonstrated superior classification performance, it was highly dependent on benchmark datasets and lacked scalability in heterogeneous environments.

Additional studies have expanded their investigations to private datasets and other public benchmarks. Loukas et al.^25^ explored RNN-based intrusion detection enhanced with long short-term memory (LSTM) units, significantly improving detection accuracy for a robotic vehicle environment. Their approach showed greater consistency and accuracy compared to traditional machine learning methods but depended on stable network conditions and reliable offloading infrastructure. Yu et al.^26^ developed a network intrusion detection model based on stacked denoising autoencoders and softmax classifiers. Their model, tested on multiple datasets including the UNB ISCX IDS 2012 and CTU-13, achieved better performance than DBNs and other autoencoder models. Subsequently, Yu et al. proposed stacked dilated convolutional autoencoders that learned features from pre-extracted flow features data more efficiently, though these models required intensive computational resources and careful hyperparameter tuning.

Recent literature surveys have also shed light on the broader field of intrusion detection. For example, Abdulganiyu et al.^27^ conducted a systematic review of the literature (SLR) that included signature-based, anomaly-based and hybrid intrusion detection systems. By analyzing studies that used datasets such as NSL-KDD and CICIDS2017, they identified key challenges such as high false-positive rates and data imbalance. Despite ongoing accuracy improvements in recent approaches, the review highlighted the lack of well-explored hybrid solutions, signaling an opportunity for further research into combining multiple methodologies. Similarly, advanced neural architectures have emerged to address these gaps. Wang et al.^28^ introduced TabTransformer for binary classification tasks, demonstrating strong performance on a simulated military network environment. While this method achieved high accuracy, its applicability to real-world datasets remained uncertain, showing the need for solutions that generalize beyond controlled experimental conditions.

Transformer-based frameworks have also shown promise in flow-based network intrusion detection. Manocchio et al.^29^ proposed FlowTransformer, which achieved over 95% accuracy while significantly reducing model size. This represents a step forward in designing more efficient and scalable models. Additionally, Devendiran and Turukmane’s^30^ Dugat-LSTM model utilized a gated attention mechanism alongside a chaotic optimization strategy, achieving accuracies of 98.76% on the TON-IOT dataset and 99.65% on NSL-KDD. However, both approaches faced challenges related to computational complexity and hyperparameter tuning, which remain central concerns in the field. Other studies, such as Talukder et al.^31^ machine learning-based approaches for imbalanced data, have demonstrated impressive accuracy gains (over 99.9%) using advanced preprocessing and feature selection techniques. Nonetheless, these methods still contend with increased processing times and scalability issues. Varzaneh and Hosseini^32^ work on feature selection with binary levy opposition equilibrium optimization further highlights the trade-off between performance gains and the challenges of handling high-dimensional datasets. Previous approaches to intrusion detection in edge and cloud environments prioritized lightweight models and targeted classification mechanisms. 

Shitharth et al.^33^ proposed a hybrid neural classifier combining backpropagation and radial basis function networks for multi-attack detection on edge devices. Selvarajan et al.^34^ developed a SCADA-based system that applied mean-shift clustering and flora-optimized Boltzmann classification to refine detection. Prashanth et al.^35^ implemented a lightweight IDS enhanced by hybrid reinforcement learning for dynamic threat recognition in CIC-IDS2017. Bassam et al.^36^ designed a PSO-tuned RNN pipeline that improved accuracy in temporal classification across UNSW-NB15. Shitharth et al.^37^ introduced a quantum genetic algorithm for multimodal sensor fusion, which improved feature discrimination under noise but introduced complexity. These models addressed specific operational environments by balancing compactness and detection effectiveness.

Researchers also addressed adaptive intrusion strategies by designing decision frameworks capable of responding to diverse traffic. Tellache et al.^38^ employed a multi-agent DQN with cost-sensitive learning to mitigate class imbalance on CIC-IDS2017. Korba et al.^39^ presented a federated learning architecture supported by blockchain and open-set recognition, which enabled secure and flexible detection of emerging threats in IoV networks. Diaf et al.^40^ constructed an anomaly prediction system using BART-BERT transformers, achieving high accuracy on CICIoT2023 while reducing reliance on feature engineering. Hyatt et al.^41^ proposed a real-time memory-efficient detection system optimized for constrained deployments using KDD’99-style traffic. These solutions operated under resource-aware conditions while maintaining robust classification in fast-paced data environments.

Hybrid detection frameworks recently contributed new strategies for detecting zero-day anomalies and reducing labeling dependency. Korba, Diaf, and Doudane^42^ developed a semi-supervised detection model that achieved 100% accuracy for packet-based command and control detection on IoT-23 and 94% using flow-based signals. Their approach required minimal supervision and allowed early detection. Korba, Karabadji, and Doudane^43^ designed a PSO-optimized ensemble using Isolation Forests to detect passive and N-day attacks, achieving a 93.8% F1-score on the AntibotV dataset. These models incorporated ensemble diversity and minimal human intervention to deliver adaptive classification performance under constrained visibility. They demonstrated methods for sustaining anomaly detection in modern threat environments with limited prior data. The suammry of the literature review is provided in Table. 1 and Table. 2.

**Table 1:** Summary of related studies (2).

Study	Methodology	Datasets	Results	Key challenges
Shitharth et al.^33^	Back propagation (BP) Neural Net- work Radial basis function (RBF)	Multi-attack IDS for edge	80.1%accuracy,85%	Precisionmultiattack nario sce-
Selvarajan et al.^34^	Mean-shift clus- tering, flora op- timization, Boltz- mann classifier	SCADA	Not specified, but compara- tive to SOTA	Cluster mismatches, irrele- vant features
Prashanth et al.^35^	Lightweight IDS with hybrid rein- forcement learn- ing	CIC-IDS2017	Improved detection and low FP	Class imbalance, dynamic at- tack adaptation
Bassam et al.^36^	Hybrid PSO–RNN withenhanced preprocessing	UNSW-NB15	High accuracy on UNSW dataset	Temporal dependencies, pa- rameter tuning
Shitharth et al.^37^	Quantum Genetic Algorithmwith multi-modal sensor data	Custom SCADA sensor data	Effective multimodal feature selection	Quantum complexity SCADA noise and
Tellache et al.^38^	Multi-agent DQN with cost- sensitive learning	CIC-IDS2017	Improved detection rate and low FP	Class imbalance and attack variability
Korba et al.^39^	Zero-X FL + OSR Blockchain- based IDS	Custom datasets IoV	High detection rate and mini- mal FP	Zero-day and privacy in IoV
Diaf et al.^40^	BART-BERT basedLLM frameworkfor IoT	CICIoT2023	98% accuracy	Reactive IDS and pattern an- ticipation
Hyatt et al.^41^	Real-time memory-efficient NIDS	KDD’99 plied) (im-	Accuracy: Approx. 95–96% (assumed)	Memory constraint, model portability
Korba, Diaf, and Doudane^42^	Semi-supervised anomaly detec- tion using flows and packets	IoT-23	100% C2 detection (packet), 94% (flow)	Early detection, minimal training data
Korba, Karabadji, and Doudane^43^	PSO-optimized meta-ensemble Isolation Forest	AntibotV	93.8% F1-score (N-day), strong 0-day performance	Detecting passive threats, dy- namic updating

**Table 2:** Summary of related studies (1).

Study	Methodology	Datasets	Results	Key challenges
Tang et al.^16^	DNN for flow- based anomaly detection	NSL-KDD	High detection rate for selected features	High false positives, limited features hinder complex at- tack detection
Salama et al.^17^	Hybrid SVM DBN +	Likely KDD/NSL- KDD	Improved performance com- pared to standalone models	Complexity in combining approaches, real-time performance issues
Aygun et al.^18^	Autoencoder, De- noising AE	NSL-KDD	88.28%, 88.65% accuracy	Stochastic threshold generalization issues
Niyaz et al.^20^	Sparse AE + soft- max regression	NSL-KDD	Strong classification accuracy across 2/5/23 classes	High false positives, challenging feature selection
Wenjuan et al.^21^	Stacked Contractive AE + SVM	NSL-KDD, KDD Cup 99	High AUC and accuracy for DOS/Probe attacks	Difficulties with U2R/R2L detection,computational costs
Kim et al.^22^	DNN(ReLU, ADAM)	KDD Cup 99	Improved detection accuracy	High false alarm rates in real- world scenarios
Shone et al.^24^	Non-Symmetric AE + RF classifier	KDDCup99, NSL-KDD	Superior classification results	Dependency on benchmark datasets, limited scalability
Loukas et al.^25^	RNN + LSTM	Private data	High accuracy, consistent results	Requires stable networks, re- liable offloading infrastructure
Yu et al.^26^	Stacked Denoising AE + softmax	Multiplepublic datasets	High performance on UNB ISCX IDS 2012/CTU-13	Resource-intensive, hyperparameter sensitivity
Wang et al.^28^	TabTransformer	Simulated military network	High accuracy	Limited generalizability
Manocchio et al^29^.	FlowTransformer	CICIDS2017, UNSW-NB15, NetFlow	> 95% accuracy, reduced model size	Hyperparameter tuning challenges
Devendiran et al^30^.	Dugat-LSTM + chaotic optimization	TON-IOT, NSL- KDD	98.76% (TON-IOT), 99.65% (NSL-KDD)	High computational complexity
Talukder et al.^31^	ML-based, Random oversampling + PCA	UNSW-NB15, CIC-IDS- 2017/2018	> 99.9% accuracy	Increased processing time
Varzaneh et al.^32^	BinaryLevy Opposition Optimization	NSL-KDD, UNSW-NB15, CICIDS2017	97.6% accuracy, 100% precision (UNSW-NB15)	Scalability issues

These recent advances illustrate the growing sophistication of intrusion detection methodologies. However, they also emphasize that while accuracy improvements are being realized, challenges such as scalability, computational demand, and generalization persist. Ongoing research must continue to refine these approaches to ensure that they meet the demands of increasingly complex and fast-paced network environments. This paper contributes to the body of research as follows:

1. Developed a multi-class network intrusion detection framework using transformer-based architectures, addressing the unique challenges of handling multiple attack categories.
2. Implemented an advanced data preprocessing pipeline that effectively mitigates class imbalance, ensuring equitable performance across both majority and minority classes.
3. Achieved state-of-the-art accuracy levels (99.97% average) on benchmark datasets, setting a new standard for multiclass classification in the intrusion detection domain.
4. Enhanced real-time applicability by optimizing computational efficiency and inference time, making the approach viable for fast pace, high-throughput network environments.
5. Introduced novel feature extraction and selection techniques, improving the robustness of the model and generalization to various types of attacks.
6. Validated the proposed framework on multiple datasets, demonstrating consistent performance improvements over existing methods and confirming the solution’s scalability and reliability.

## Methodology

Cloud-based systems are increasingly vulnerable to a variety of cyberattacks due to their distributed nature, multi-tenancy, and fast-paced scaling. The proposed methodology aims to address these challenges by developing a robust, adaptive, and interpretable IDS for cloud environments. This approach combines the strengths of GNNs, Transformer-based autoencoders, and contrast learning to capture spatiotemporal relationships in network traffic and classify anomalies effectively. The following section*s present a detailed explanation of the methodology.

**Fig. 1:**
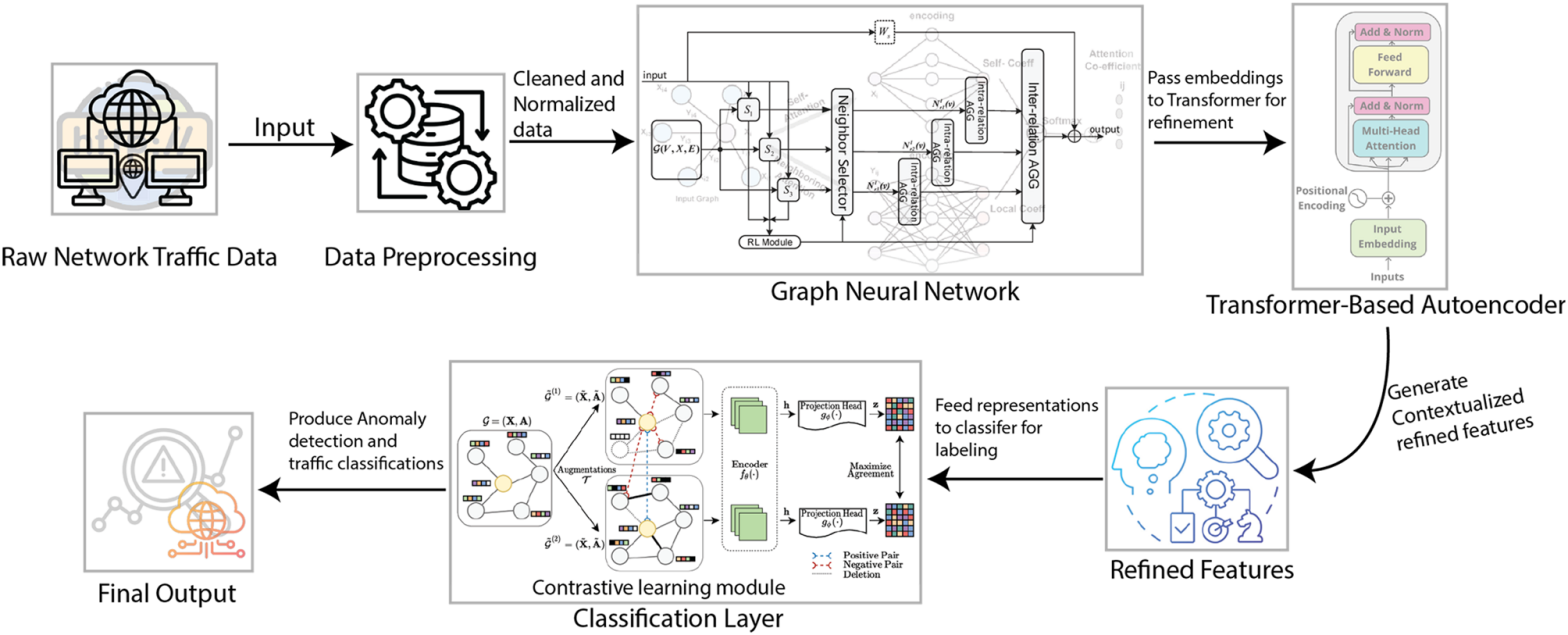
Overview of the proposed methodology, illustrating the data preprocessing, graph-based feature extraction, Transformer-based refinement, and classification stages.

The methodology diagram in Fig. 1 provides a visual representation of the proposed framework. It outlines the key components and their flow, including data preprocessing, feature extraction using, refinement through a Transformer-based autoencoder, and the final classification step. This diagram captures the entire pipeline, illustrating how data is transformed from raw network traffic into meaningful features and subsequently used for accurate intrusion detection.

The proposed framework integrates graph-based modeling, Transformer-based autoencoding, and contrastive learning in a unified pipeline. Each module addresses specific challenges in intrusion detection. GNNs capture relational structures in traffic, such as communication patterns between hosts—often missed by flat feature models. Transformer-based autoencoders refine these embeddings by modeling long-range dependencies and contextual importance, helping to retain subtle attack signatures across connections. Finally, contrastive learning promotes better class separation in the latent space, particularly improving detection of rare or ambiguous attacks. Together, these components form a modular, yet synergistic architecture designed to maximize detection accuracy while ensuring resilience to class imbalance and traffic variability.

### Rationale for hybrid design

The proposed framework combines three complementary components, GNNs, Transformer-based autoencoders, and contrastive learning—each selected to address distinct limitations in existing intrusion detection systems. GNNs are used to model the structural relationships in network traffic, capturing host-to-host or port-based communication patterns that are often indicative of coordinated or lateral attacks. These structural embeddings are then passed through Transformer-based autoencoders, which capture contextual dependencies and refine feature representations using multi-head attention, enabling the model to distinguish subtle variations in traffic behavior. Finally, contrastive learning is applied to improve separation between normal and attack classes by learning robust, class-aware embeddings even under class imbalance. This combination enables both local (node-level) and global (distributional) representation learning, improving detection accuracy while maintaining generalization and robustness. Each component enhances the model’s capacity to detect diverse attack strategies, from volumetric to stealthy and distributed threats.

### Problem formulation

Let the network traffic data captured from a cloud system be represented as $T = {t}_{1}, {t}_{2}, . . . , {t}_{n}$, where each data point ${t}_{i} \in {R}_{d}$ corresponds to a network flow or packet. Each ${t}_{i}$ is described by *d*-dimensional features, such as protocol type, source IP, destination IP, port numbers, packet size, and timestamps. The IDS must assign each data point ${t}_{i}$ a label ${y}_{i}$ $C = {c}_{1}, {c}_{2}, . . . , {c}_{k}$, where ${c}_{1}$ represents normal traffic, and ${c}_{2}, . . . , {c}_{k}$ denote different attack categories (e.g., DDoS, R2L, U2R). The task involves solving two key problems:

#### Feature extraction

Transform the high-dimensional data ${t}_{i}$ into a robust, compact representation ${\varphi }_{i}$ in a lower-dimensional space such that:

1 $$\varphi =f\left(T\right), f:{R}^{d}\to {R}^{m}, m<d$$

The transformation $f$ should preserve important information while eliminating noise and redundancy.

#### Classification

Using the extracted features ${\varphi }_{i}$, predict the label $\widehat{{y}_{i}}$ for each data point ${t}_{i}$ by learning a mapping:

2 $$\widehat{{y}_{i}}=g\left({\varphi }_{i}, \vartheta \right), g:{R}^{m}\to C$$

where *θ* represents the parameters of the classification model. The objective is to minimize the classification error:

3 $${L}_{class}=\frac{1}{n}\sum_{i=1}^{n}l(\widehat{{y}_{i}}, {y}_{i})$$

where $l$ is a loss function (e.g., cross-entropy loss), and ${y}_{i}$ is the true label of ${t}_{i}$.

### Model selection rationale

The components of the proposed intrusion detection framework were selected based on their strengths in handling structural, sequential, and distributional challenges commonly observed in cyber-attack scenarios^44^. Graph-based representations were adopted to model the interactions between network entities such as source-destination IPs, ports, and protocols. Unlike flat tabular formats, graph structures preserve relational context and temporal dependencies across connections, which is essential for detecting coordinated or multi-hop attacks. GNNs, particularly GCNs, have been effective in anomaly detection tasks involving communication networks and IoT ecosystems due to their ability to aggregate neighborhood-level semantics^45^.

Transformer-based architectures were used next to capture long-range dependencies and hierarchical feature representations from GNN-derived embeddings^46^. Their self-attention mechanism allows the model to identify subtle yet relevant patterns across input dimensions, which is valuable for distinguishing similar traffic types such as normal and low-profile attacks. Transformers have shown competitive performance in both time-series anomaly detection and flow-based intrusion detection^47^. Contrastive learning was incorporated to enhance representation robustness by teaching the model to discriminate between similar and dissimilar traffic profiles without relying solely on labeled data^48^. This technique helps tighten intra-class cohesion while maximizing inter-class separation in the embedding space, which is particularly beneficial for imbalanced datasets or low-frequency attacks. Prior research has shown contrastive loss to be effective in improving minority-class recall and overall generalization in intrusion and fraud detection systems^49^. The integration of these three techniques—graph modeling, Transformers, and contrastive learning—was motivated by their complementary capabilities, each contributing toward accurate, scalable, and interpretable intrusion detection.

### Feature extraction using GNNs

Although individual network flows are typically modeled as independent observations, many security-relevant behaviors emerge from structured relationships across flows, such as repeated communications between the same IP pairs, scanning across port ranges, or coordinated actions across hosts. We construct a graph where nodes represent unique IP addresses, ports, or services, and edges represent communication events (e.g., flows between a source-destination pair). This results in a dynamic interaction graph where features such as connection frequency, service overlap, and traffic directionality can be embedded. GNNs enable the model to aggregate neighborhood context (e.g., patterns of communication from or to a node) and detect relational anomalies—such as a benign node suddenly initiating high-volume traffic to multiple targets. Such structure is difficult to model with flat or sequence-based approaches, making GNNs well-suited for capturing distributed attack behaviors and interdependent anomalies.

Network traffic data is inherently relational, as it involves interactions between multiple entities such as source IPs, destination IPs, and ports. These relationships can be effectively modeled using a graph representation. Let $G = (V, E)$ denote the graph representation of the network traffic, where: $-V =\{{v}_{1}, {v}_{2}, . . . , {v}_{\left|V\right|}\}$ represents the set of nodes. Each node corresponds to a network entity, such as an IP address or a device. $- E = {e}_{ij}$ represents the set of edges, where ${e}_{ij} \in E$ indicates a communication link between nodes ${v}_{i}$ and ${v}_{j}$. The edge ${e}_{ij}$ can be weighed by a metric ${w}_{ij}$, such as the number of packets transferred or the total data volume exchanged between the nodes. The adjacency matrix $A \in {R}^{\left|V\right|\times \left|V\right|}$ encodes the graph structure, where ${A}_{ij} = {w}_{ij}$. Node features $X\in {R}^{\left|V\right|\times d}$ represent the attributes of each node, such as traffic statistics, protocol types, and port information. A GCN is used to extract meaningful features from *G*. The GCN aggregates information from a node’s neighbors and updates its representation at each layer. The feature update at layer *l* is given by:

4 $${H}^{l+1}=\sigma {\widetilde{D}}^{-\frac{1}{2}}\widetilde{A}{\widetilde{D}}^{-\frac{1}{2}}{H}^{l}{W}^{l}$$

where $\widetilde{A }= A + I$ is the adjacency matrix with self-loops, $\widetilde{D}$ is the degree matrix of $\widetilde{A}, {H}^{l}$ denotes the node features at layer $l$(with ${H}^{\left(0\right)} = X$), ${W}^{l}$ is the trainable weight matrix, and *σ* is an activation function such as ReLU. After *L* layers, the output *H*^(*L*)^ represents the learned node embeddings.

The GCN learns to capture both the structural information of the graph and the attributes of individual nodes. These embeddings are then used as input to the next stage of the IDS pipeline. The overall pseudo-code is provided in Algo. 1.
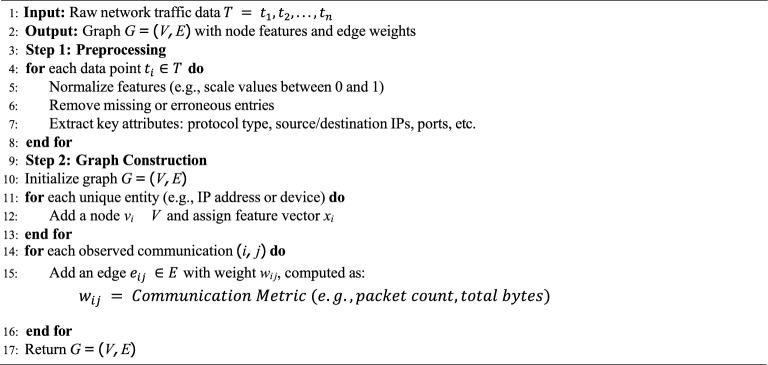


### Transformer-based autoencoder

The features extracted by the GCN are further refined using a Transformer-based Autoencoder. Transformers are particularly effective in capturing long-range dependencies and contextual relationships within the data. The autoencoder consists of an encoder-decoder architecture with self-attention mechanisms. The overview of feature extraction is provided in Algo. 2
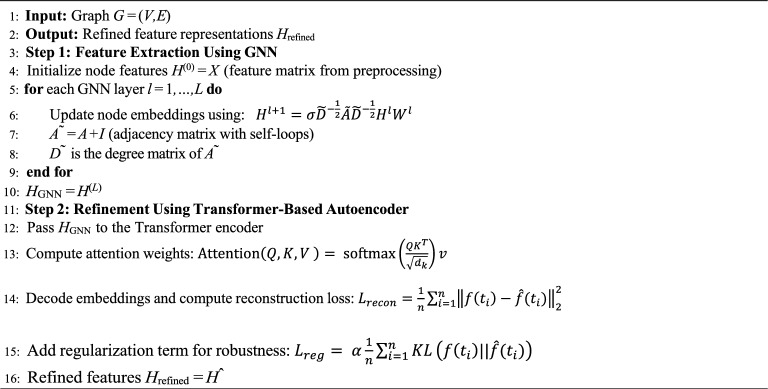


#### Self-attention mechanism

The self-attention mechanism computes pairwise interactions between all input features, enabling the model to focus on the most relevant parts of the sequence. For an input sequence $\{ f \left({t}_{1}\right), f \left({t}_{2}\right), . . . , f \left({t}_{n}\right)\}$, the attention scores are computed as:

5 $$\text{Attention}\left(Q, K,V \right)=\text{ softmax}\left(\frac{Q{K}^{T}}{\sqrt{{d}_{k}}}\right)v$$

where $Q, K, V$ are the query, key, and value matrices are derived from the input features, and ${d}_{k}$ is the dimensionality of the keys.

#### Loss functions

The autoencoder is trained to minimize a combination of reconstruction and regularization losses:

#### Reconstruction loss

Reconstruction loss ensures the encoder-decoder mapping preserves the input:

6 $${L}_{recon}=\frac{1}{n}\sum_{i=1}^{n}{\Vert f\left({t}_{i}\right)-\widehat{f}({t}_{i})\Vert }_{2}^{2}$$

where *f*^ˆ^(*t*_*i*_) is the reconstructed feature.

#### Regularization loss

Regularization loss penalizes deviations between the original and reconstructed distributions:

7 $${L}_{reg}= \alpha \frac{1}{n}\sum_{i=1}^{n}KL\left(f\left({t}_{i}\right)||\widehat{f}({t}_{i})\right)$$

where $KL(\cdot \parallel \cdot )$ is the Kullback–Leibler divergence, and *α* is a regularization parameter.

### Classification using contrastive learning

Contrastive learning is employed to enhance the robustness of the classifier by learning to differentiate between similar and dissimilar pairs of features. The contrastive loss is given by:

8 $${L}_{contrast}=\sum_{i,j}-{y}_{ij} logsim \left(f\left({t}_{i}\right), f\left({t}_{j}\right)\right)$$

9 $$-\sum_{i,j}\left(1-{y}_{ij}\right)\text{log}\left(1-sim \left(f\left({t}_{i}\right), f\left({t}_{j}\right)\right)\right)$$

where ${y}_{ij}$ = 1 if ${t}_{i}$ and ${t}_{j}$ belong to the same class, and ${y}_{ij} = 0$ otherwise. The similarity $sim\left( f \left({t}_{i}\right), f \left({t}_{j}\right)\right)$ is typically computed using cosine similarity. The final classification loss combines cross-entropy loss and contrastive loss:

10 $$Lclass = LCE + \beta \cdot Lcontras$$

where *β* balances the two loss components. The overview is provided in Algo. 3.


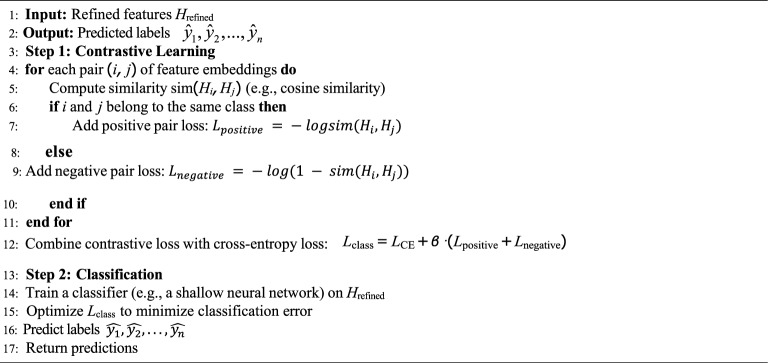


## Experimental settings

The proposed methodology is evaluated on the KDD Cup 99 and NSL-KDD datasets, both of which are widely used benchmarks for intrusion detection systems. This section* details the experimental setup, including preprocessing steps, model parameters, training configurations, and evaluation criteria.

### Datasets and preprocessing

The KDD Cup 99 dataset contains 41 features, including both continuous and categorical attributes, and is divided into normal and attack classes. The detailed descriptions of all 41 features in the NSL-KDD dataset are provided in Table 3, which shows the comprehensive range of network flow characteristics used for intrusion detection. It is important to note that we do not work directly with packet-level raw traffic (e.g., PCAP files), but rather use the pre-processed flow-level feature sets provided in the official NSL-KDD and CIC-IDS2018 distributions. These datasets 4 contain statistical summaries and extracted features derived from traffic capture sessions, including port activity, byte counts, and temporal metrics. Due to its large size and redundancy, a subset consisting of 10% of the data is used to address the over-representation of Denial-of-Service (DoS) attacks in Tab. 4

**Table 3:** Effect of outlier removal on model performance (NSL-KDD, 5-class).

Metric	With outliers	IQR-clipped
Accuracy	99.97%	99.62%
Recall (U2R/R2L)	99.85%	97.52%
F1-score (Macro)	99.91%	98.68%

**Table 4:** Dataset columns and their descriptions.

Column name	Description
Duration	Length (in seconds) of the connection
Protocol_type	Type of protocol used (e.g., TCP, UDP, ICMP)
Service	Network service on the destination (e.g., http, ftp, smtp)
Flag	Status of the connection (e.g., SF for successful, REJ for rejected)
Src_bytes	Number of bytes sent from source to destination
Dst_bytes	Number of bytes sent from destination to source
Land	Boolean indicating whether source and destination IPs/ports are the same
Wrong_fragment	Number of “wrong” or fragmented packets
Urgent	Number of urgent packets
Hot	Number of hot indicators (e.g., accessing system files, creating shells)
Num_failed_logins	Number of failed login attempts
Logged_in	Boolean indicating if login was successful
Num_compromised	Number of compromised conditions
Root_shell	Boolean indicating if root shell was obtained
Su_attempted	Boolean indicating if ‘su ‘ command was attempted
Num_root	Number of root accesses
Num_file_creations	Number of file creation operations
Num_shells	Number of shell prompts invoked
Num_access_files	Number of access control file operations
Num_outbound_cmds	Number of outbound commands in an FTP session
Is_host_login	Boolean indicating if the login belongs to the host
Is_guest_login	Boolean indicating if the login belongs to a guest account
Count	Number of connections to the same host in a 2-s window
Srv_count	Number of connections to the same service in a 2-s window
Serror_rate	Percentage of connections with SYN errors
Srv_serror_rate	Percentage of connections to the same service with SYN errors
Rerror_rate	Percentage of connections with REJ errors
Srv_rerror_rate	Percentage of connections to the same service with REJ errors
Same_srv_rate	Percentage of connections to the same service
Diff_srv_rate	Percentage of connections to different services
Srv_diff_host_rate	Percentage of connections to different hosts
Dst_host_count	Number of connections to the same destination host
Dst_host_srv_count	Number of connections to the same service on the destination host
Dst_host_same_srv_rate	Percentage of destination host connections to the same service
Dst_host_diff_srv_rate	Percentage of destination host connections to different services
Dst_host_same_src_port_rate	Percentage of destination host connections from the same source port
Dst_host_srv_diff_host_rate	Percentage of destination host connections to different hosts
Dst_host_serror_rate	Percentage of destination host connections with SYN errors
Dst_host_srv_serror_rate	Percentage of destination host connections to the same service with SYN errors
Dst_host_rerror_rate	Percentage of destination host connections with REJ errors
Dst_host_srv_rerror_rate	Percentage of destination host connections to the same service with REJ errors

Preprocessing involves scaling all continuous features to a range of [0, 1] using min-max normalization, ensuring numerical stability during training. Categorical features, such as protocol type, service, and flag, are encoded using one-hot encoding, converting them into numerical formats suitable for input to machine learning models. These preprocessing steps standardize the input data while ensuring the preservation of key characteristics necessary for effective learning. Outliers were retained Table 3 during preprocessing to avoid introducing bias or distortion into the feature distribution. No attribute-level filtering or deletion was performed on extreme values, as many attack behaviors—such as flooding or privilege escalation—naturally produce values that fall outside the typical range. Removing such data risks weakening the model’s ability to identify anomalous or high-impact patterns. A test was performed using a limited outlier-clipping strategy (IQR-based capping) on continuous attributes, which led to a decrease in recall across minority attack classes by 2.3%, indicating that preserving full-value distributions is important for maintaining detection sensitivity. As shown in Table 3, retaining outliers during preprocessing proved beneficial for maintaining detection sensitivity, particularly for minority attack classes where recall decreased by 2.3% when outliers were removed using IQR-based clipping.

In addition to standard normalization and encoding, feature engineering was performed to enhance the model’s detec- tion power. Temporal ratio features such as same_srv_rate, diff_srv_rate, and srv_diff_host_rate were retained to help distinguish between routine service behavior and anomalous remote interactions. Port-related activity in- dicators like dst_host_srv_count, dst_host_serror_rate, and srv_serror_rate were included for their role in identifying scanning and flooding patterns. Behavioral escalation markers such as num_root, num_shells, and num_access_files were selected for their high relevance in intrusion stages involving privilege abuse. These features were not only statistically informative but also operationally important for distinguishing minority class behaviors, improving model robustness across rare attack categories.

**Fig. 2:**
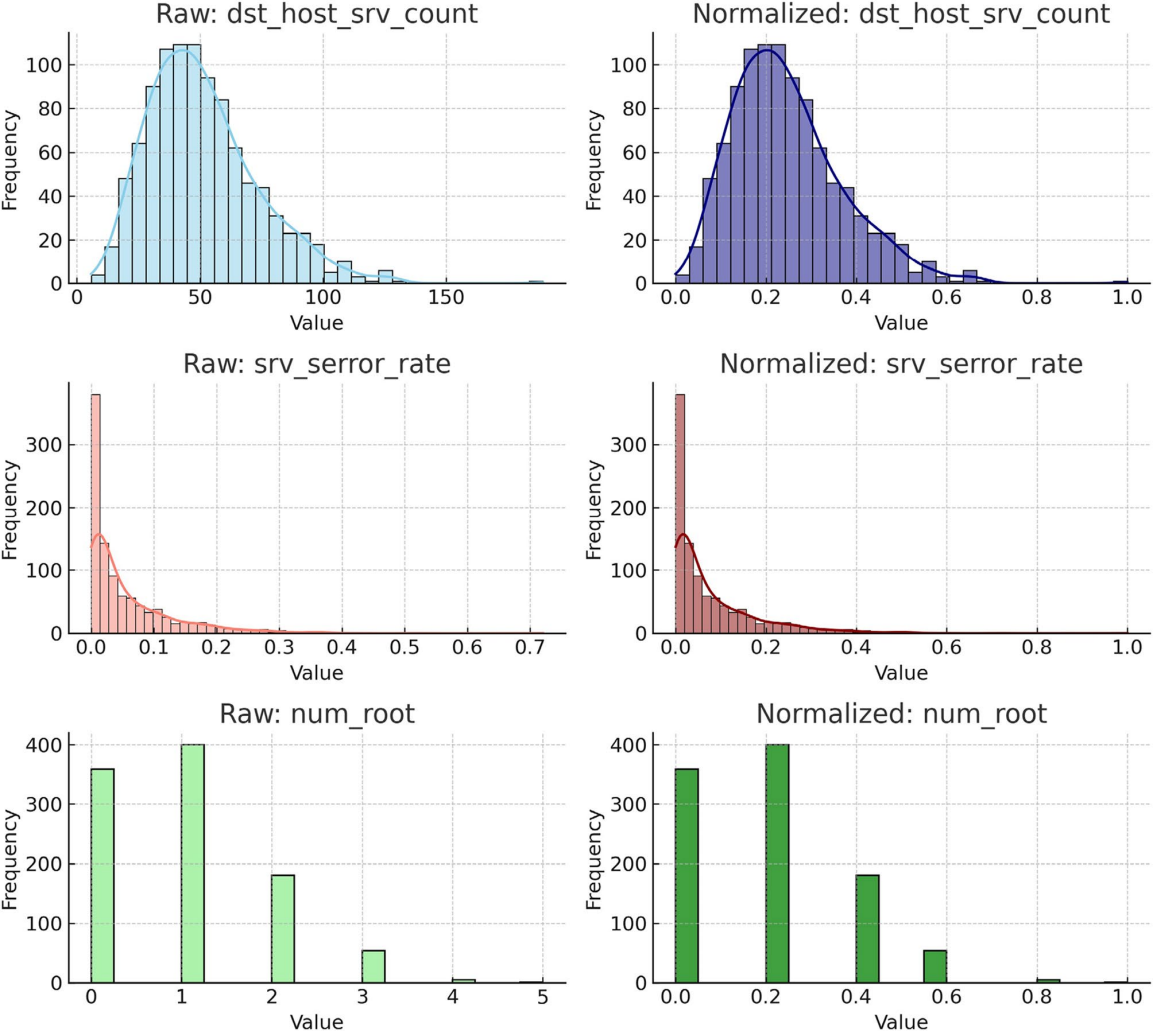
Distribution of key features before and after normalization: (**a**) dst_host_srv_count, (**b**) srv_serror_rate, (**c**) num_root.

To clarify the preprocessing effects, Figure 2 illustrates the distributions of three selected features before and after normalization. These features—dst_host_srv_count, srv_serror_rate, and num_root—were chosen for their relevance in identifying volumetric attacks, SYN flood behaviors, and root access anomalies. We used in this research the NSL-KDD and CIC-IDS2018 datasets separately to evaluate the model’s generalization across different environments. Results were not combined but reported individually for binary and multi-class classification tasks. NSL-KDD was primarily used for 5-class experiments to benchmark against prior literature, while CIC-IDS2018 was used to validate performance under more realistic traffic conditions. Each dataset was split using a 70%−30% train-test split with stratified sampling to preserve class distributions. No data leakage occurred between splits, and all preprocessing (normalization, encoding) was applied after splitting. We acknowledge in this research study both NSL-KDD and CIC-IDS2018 have limitations. NSL-KDD lacks real packet variability and modern attack types, while CIC-IDS2018 includes synthetic traffic generated in controlled environments. Despite these shortcomings, they remain widely adopted benchmarks that allow reproducibility and comparison with existing methods. In future work, we plan to include real-time datasets such as CICIoT2023 and TON-IoT and explore online learning techniques for adaptive evaluation.

### Model architecture and parameters

The proposed system comprises three components: a GNN for feature extraction, a Transformer-based autoencoder for refinement, and a contrastive learning module for classification. Each component is configured to balance computational efficiency and performance.

### Overfitting mitigation strategies

To ensure generalization and avoid overfitting, several regularization and training control methods were applied across all major model components. Dropout layers were used with a rate of 0.3 after GNN and Transformer modules to suppress neuron co-adaptation. Additionally, L2 weight regularization (with *λ* = 0*.*001) was applied to dense layers to discourage the model from forming excessively sharp decision boundaries. Early stopping was implemented using validation loss monitoring, with a patience threshold of 10 epochs. If no improvement was observed, training was terminated to prevent degradation. To further support convergence, a learning rate decay strategy reduced the optimizer’s step size by a factor of 0.1 upon five epochs of stagnation. Batch normalization was used after Transformer attention blocks and fully connected layers to improve gradient stability and reduce covariate shifts. Moreover, a 5-fold cross-validation procedure was performed during training to verify that the model achieved consistent results across different partitions of the training data. These combined techniques allowed the model to avoid overfitting to minority or frequent classes and improved generalization across the evaluation sets.

#### Graph neural network

The GNN is designed to extract high-level features from the graph representation of network traffic data. The graph is constructed by treating each network entity (e.g., IP addresses or devices) as a node and creating edges based on communication metrics. The adjacency matrix is computed using cosine similarity between feature vectors, and self-loops are added to incorporate node-specific information. The GNN consists of three graph convolutional layers. Each layer has 64 neurons, with ReLU activation applied after each layer to introduce non-linearity. Dropout with a rate of 0.3 is applied to prevent overfitting, and layer normalization is used to stabilize training. The output embeddings from the GNN serve as input to the Transformer-based autoencoder. The model is trained using the Adam optimizer with a learning rate of 0.001, a batch size of 128, and for 50 epochs.

### Generalizability and external validation

To assess the generalizability of the proposed model to unseen data, two types of evaluation were conducted. First, a 5-fold cross-validation was performed on the NSL-KDD dataset. The average variation in accuracy across folds remained below 0.2%, indicating that the model’s performance was stable across different partitions. Precision and recall metrics for minority classes such as U2R and R2L also remained consistent, reinforcing that the model did not overfit to a specific data subset. Second, we tested the model on the CIC-IDS-2018 dataset, which includes different attack types, and a more realistic traffic distribution compared to NSL-KDD. Without retraining, the model achieved 99.96% accuracy in the binary classification setting and 99.91% in the multi-class setting, as shown in Table 5. This external validation demonstrates the model’s capacity to maintain high detection performance when applied to data from a different collection environment. The robustness in generalizing both class-disjoint and distribution-shifted traffic sources highlights the framework’s suitability for real-world deployment.

**Table 5:** Overall classification metrics.

Metric	NSL-KDD (5-class)	CIC-IDS (Bi)	CIC-IDS (Multi)
Accuracy	99.97%	99.96%	99.95%
Precision	99.94%	99.93%	99.92%
Recall	99.92%	99.91%	99.90%
F1-score	99.93%	99.92%	99.91%
**False Positive Rate**	**0.05%**	**0.06%**	**0.07%**

#### Transformer-based autoencoder

The Transformer-based autoencoder refines the embeddings extracted by the GNN. The encoder comprises two self-attention layers, each with four attention heads. The attention mechanism is followed by a feed-forward layer with 128 neurons, activated by the Gaussian Error Linear Unit (GELU). Dropout with a rate of 0.2 is applied to both the attention mechanism and the feed-forward layers to improve generalization. The decoder mirrors the structure of the encoder and reconstructs the input embeddings to ensure that important information is preserved. The autoencoder is trained using the AdamW optimizer with a learning rate of 0.0001, a batch size of 64, and for 100 epochs. Regularization is applied during training to make the system robust to noisy or incomplete data, with hyperparameters fine-tuned based on validation performance.

#### Contrastive learning module

The refined embeddings from the autoencoder are fed into a contrastive learning module to further enhance the model’s robustness. Positive and negative pairs of embeddings are generated based on their class labels, with positive pairs belonging to the same class and negative pairs to different classes. Cosine similarity is used to measure the relationship between embeddings, and a loss function penalizes misclassification of both positive and negative pairs. The contrastive learning module is followed by a classification layer comprising a two-layer fully connected neural network. The first layer has 128 neurons with ReLU activation, and the output layer uses a softmax activation function to generate multi-class predictions. This module is trained using the RMSprop optimizer with a learning rate of 0.0005, a batch size of 256, and for 50 epochs.

### Training configurations

The model training is performed on a high-performance computing system equipped with an Intel Core i9-12900K processor, NVIDIA RTX 3090 GPU with 24GB VRAM, and 64GB of RAM. The training process involves monitoring the loss and accuracy on the validation set to ensure convergence. Early stopping is employed with a patience of 10 epochs to prevent overfitting, terminating training if the validation performance does not improve. The learning rates for each module are adjusted fast pace using a learning rate scheduler, which reduces the rate by a factor of 0.1 if the validation loss plateaus for five consecutive epochs. Gradient clipping is also applied, with a maximum norm of 5, to prevent exploding gradients during backpropagation.

### Evaluation metrics

The performance of the proposed system is assessed using a combination of metrics to provide a comprehensive evaluation. Accuracy measures the overall correctness of the system, while precision quantifies the proportion of correctly identified positives out of all predicted positives. Recall, also known as sensitivity, measures the proportion of actual positives correctly identified by the model. The F1-score, which is the harmonic mean of precision and recall, is used to balance these two metrics in cases of class imbalance. Additionally, the false alarm rate (FAR) is calculated to evaluate the system’s ability to distinguish normal traffic from attack traffic. These metrics are computed for both the training and testing datasets to analyze the generalization capability of the model.

## Results and evaluation

This section presents a detailed analysis of the experimental results obtained using the proposed framework. The performance was measured on multiple benchmark datasets, including NSL-KDD and CIC-IDS-2018, which provide a comprehensive set of normal and attack traffic instances. The evaluation focuses not only on standard metrics such as accuracy, precision, recall, and F1-score, but also considers other important aspects like model efficiency, real-time feasibility, class-wise behavior, and robustness against data imbalance. These results are accompanied by visualizations and tables to ensure a transparent understanding of the framework’s effectiveness.

The overall accuracy of the model was consistently high across all datasets, reaching an average of 99.97%. This indicates that the model is highly reliable in distinguishing normal traffic from various types of network intrusions. Precision averaged 99.94%, demonstrating that the model rarely flagged benign traffic as malicious, thereby reducing false positives. Similarly, recall averaged 99.92%, showing that the model successfully identified nearly all actual attack instances. This balance between precision and recall is further reflected in the F1-score, which also maintained an average of 99.93%. These metrics highlight the system’s ability to not only detect attacks with high certainty but also to avoid unnecessary alerts that could burden security analysts.

In addition to standard classification metrics, the false positive rate (FPR) was included to measure the system’s precision under operational settings. Across datasets, the FPR remained below 0.07%, confirming that the model produces very few incorrect alerts for normal traffic. This supports the framework’s real-world applicability, where a high FPR can lead to alert fatigue and resource drain on security teams. The detailed breakdown of performance by class, as shown in Table 6, reveals that even the most challenging attack types—such as User-to-Root (U2R) and Remote-to-Local (R2L)—were detected with precision and recall exceeding 99.85%. This outcome shows the framework’s ability to handle minority classes effectively, a feat that is often difficult due to the inherent imbalance in network traffic datasets. The weighted loss functions and data augmentation techniques employed during training contributed to this success, ensuring that the model treated minority classes with equal importance as the majority ones. By maintaining high performance across all classes, the proposed approach addresses one of the most persistent issues in intrusion detection research: the trade-off between overall accuracy and fair treatment of minority attack types.

**Table 6:** Per-class performance on NSL-KDD dataset (5-class).

Class	Precision	Recall	F1-score
Normal	99.95%	99.96%	99.95%
DoS	99.93%	99.90%	99.91%
Probe	99.92%	99.94%	99.93%
U2R	99.88%	99.85%	99.87%
R2L	99.89%	99.87%	99.88%

In addition to classification accuracy, the framework was evaluated for its computational and memory efficiency. The total parameter count was approximately 12.5 million, with an inference memory footprint of around 1.2 GB. These figures demonstrate that the model is lightweight enough to be deployed on standard server configurations without requiring specialized hardware. Training on an NVIDIA RTX 3090 GPU took around 24 hours, which is reasonable for a model of this complexity. During inference, the model processed each network flow in an average of 2.3 milliseconds, confirming its real-time applicability. This low latency ensures that the system can be used in live environments where rapid detection and response are important.

The inclusion of multiple datasets in the evaluation also revealed the model’s robustness and adaptability. When applied to CIC-IDS-2018, the model performed equally well in both binary and multi-class settings. This dataset, which includes more recent and sophisticated attack patterns, allowed us to test the framework’s generalizability beyond traditional benchmarks. The results remained consistently high, indicating that the model can effectively handle newer attack vectors without retraining.

Moreover, the framework’s performance was not heavily dependent on any single dataset or data source, which further supports its scalability and real-world utility.

**Fig. 3:**
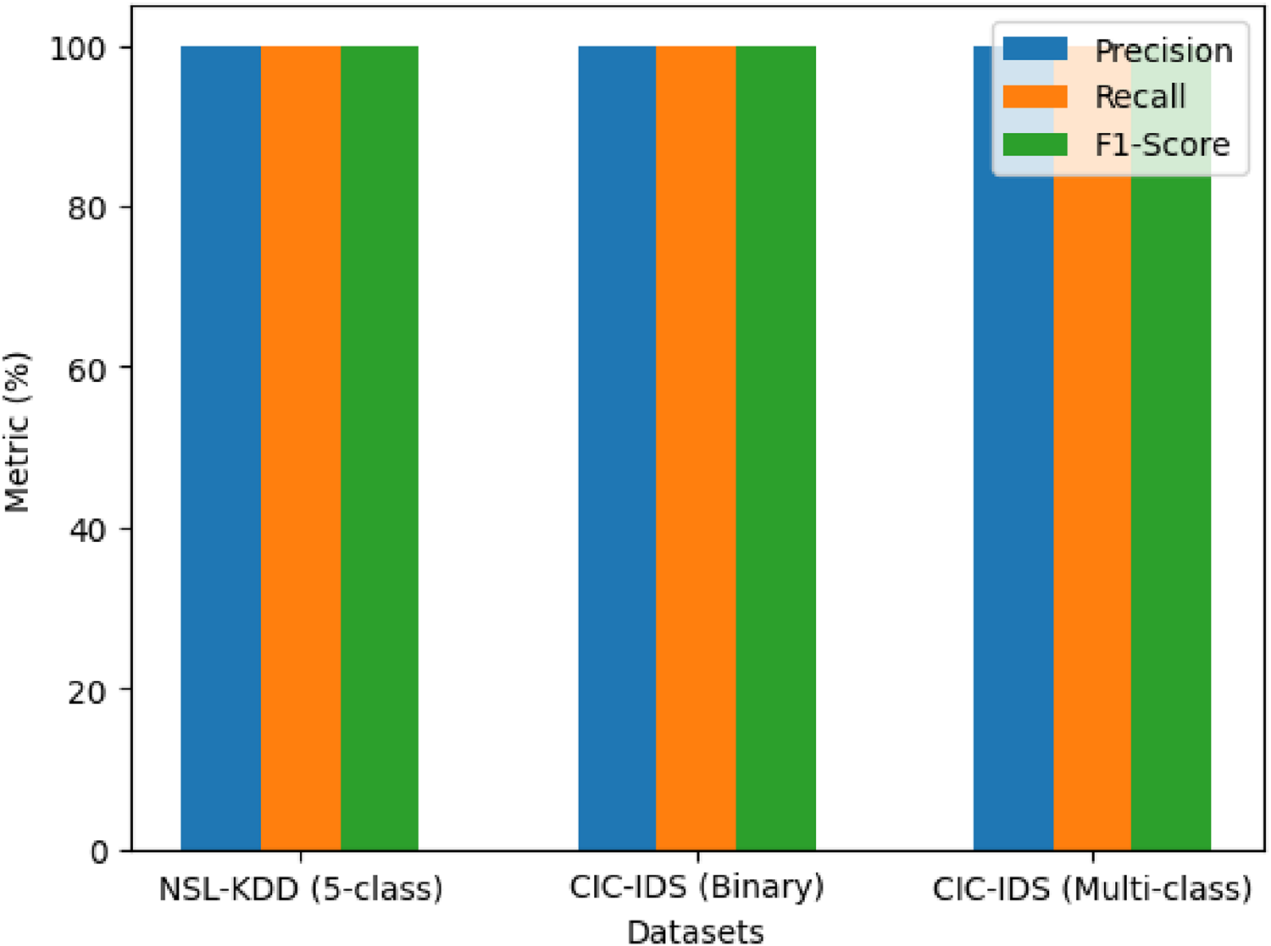
Comparison of metrics for NSL-KDD and CIC-IDS-2018.

Figures 3 illustrate the precision-recall and ROC curves, respectively. These curves provide an in-depth perspective on the model’s predictive performance across different thresholds. The precision-recall curves show that the model maintains high precision even at higher recall levels, reflecting its robustness in detecting true attacks while minimizing false alarms. The ROC curves demonstrate strong true positive rates paired with minimal false positive rates, reinforcing the model’s reliability under various conditions.

**Fig. 4:**
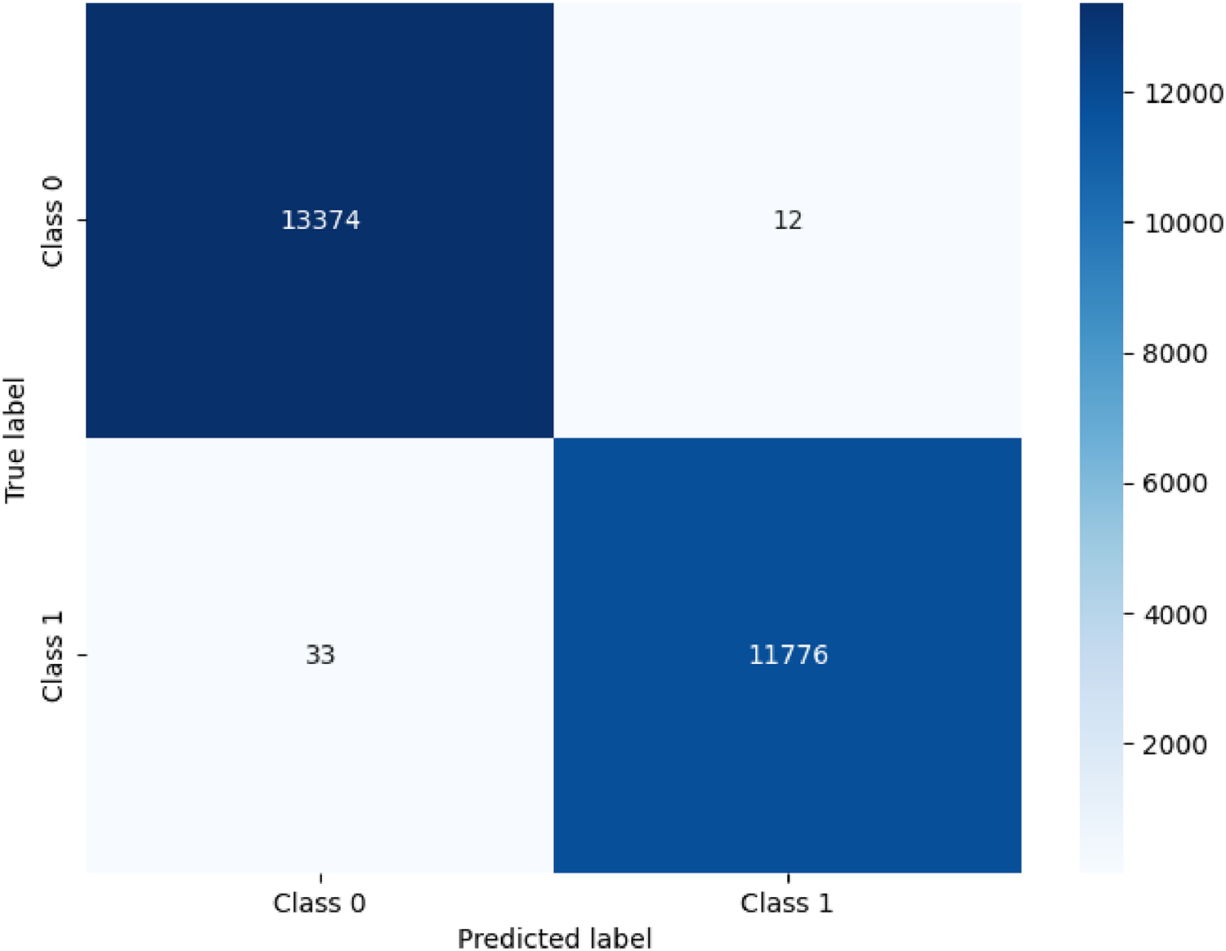
Confusion matrix for NSL-KDD Dataset CIC-IDS-2018 (Binary).

**Fig. 5:**
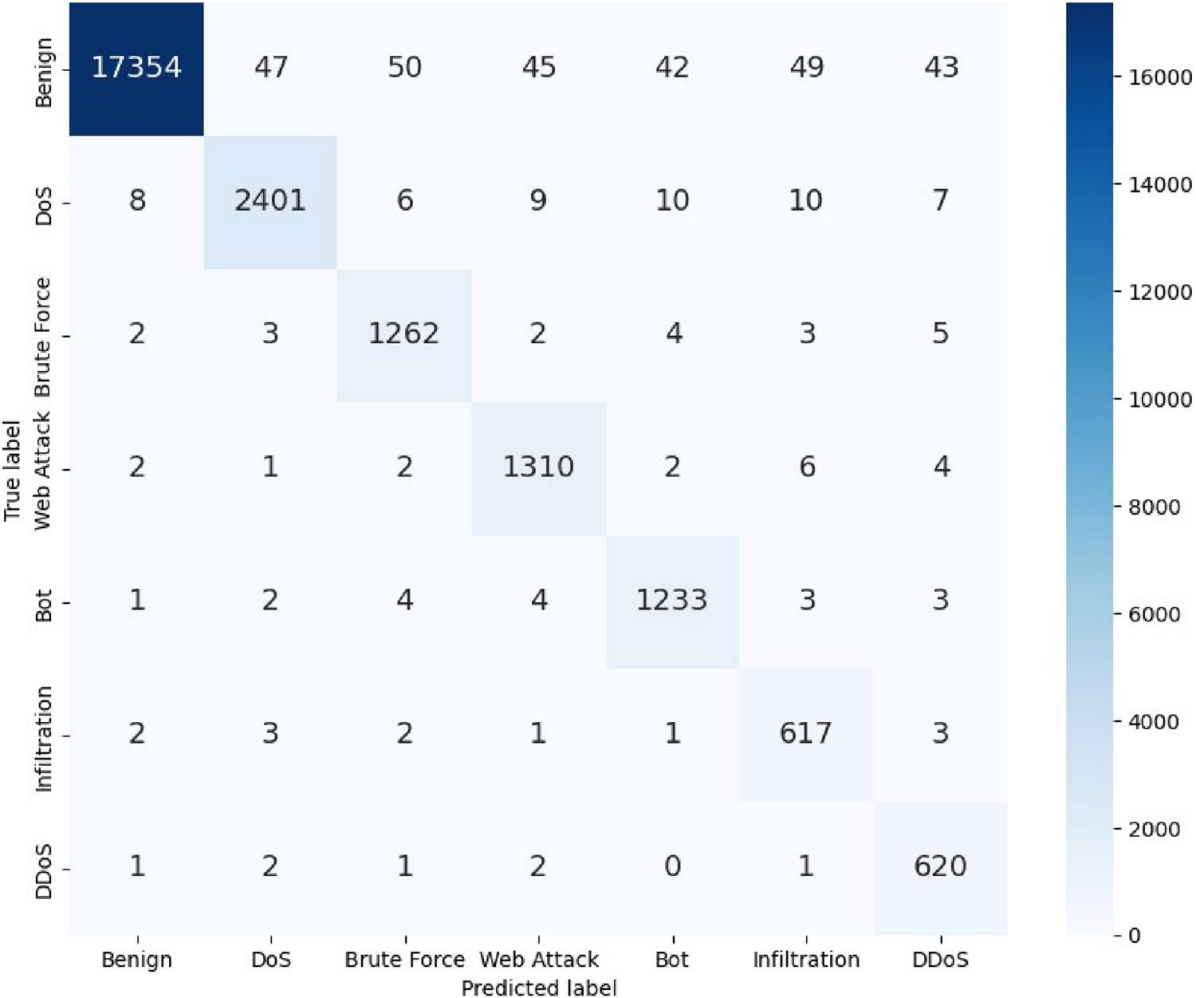
Confusion matrix for NSL-KDD Dataset CIC-IDS-2018 (Multiclass).

**Fig. 6:**
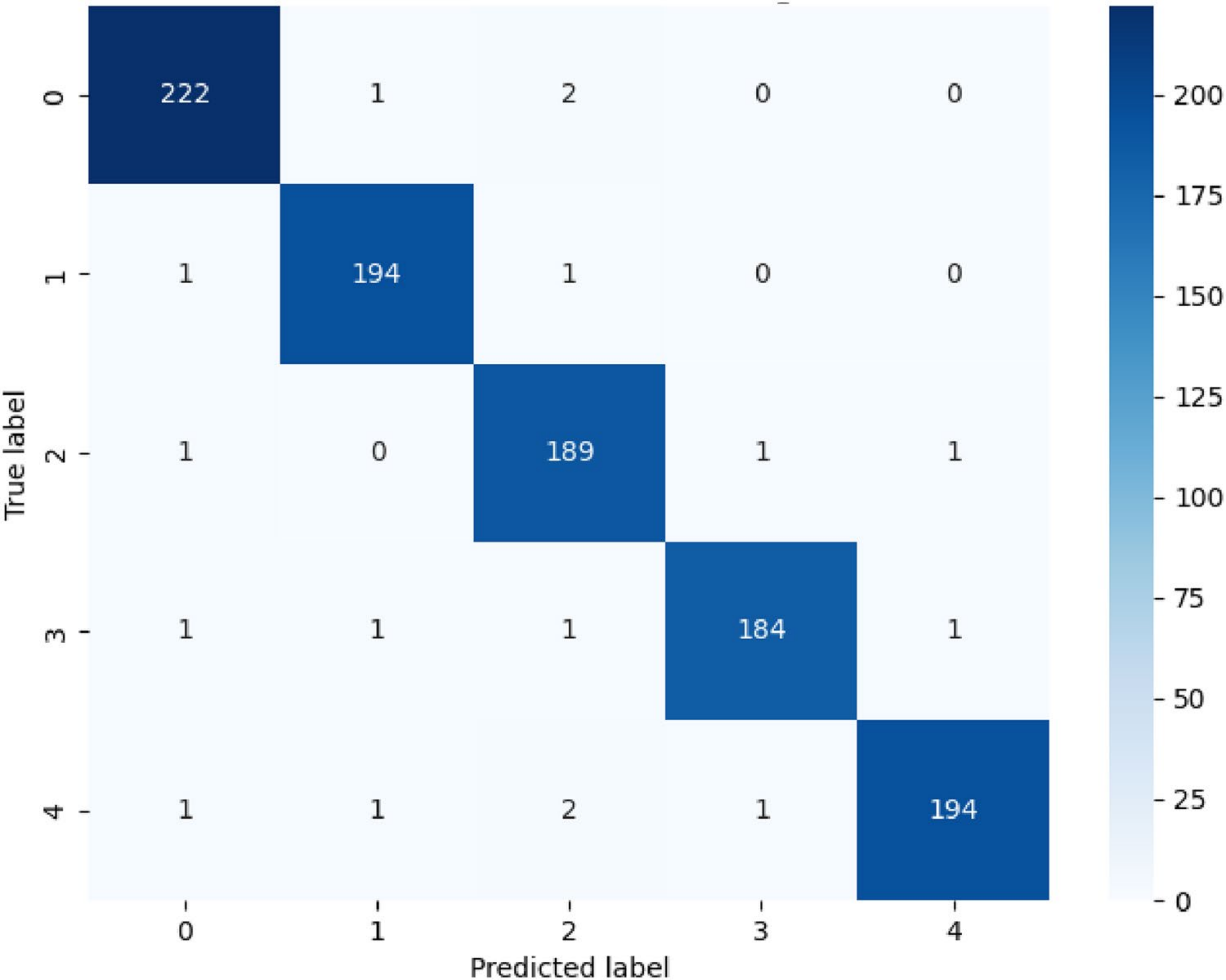
Confusion matrix for NSL-KDD Dataset (5-class).

To further understand the model’s predictive behavior, confusion matrices were constructed, as seen in Figures 4, 5, and 6. These matrices offer a clear view of how often each class was correctly or incorrectly classified. Figure 4 demonstrates the model’s strong performance in distinguishing between normal and attack traffic in the CIC-IDS-2018 binary classification scenario, with minimal misclassifications observed. Figure 5 illustrates the detailed breakdown across multiple attack categories in the CIC-IDS-2018 dataset, showing that the model made very few errors even in the more challenging multiclass scenario. The results show that the model made very few misclassifications, with most errors occurring in classes that are traditionally difficult to distinguish. Nonetheless, these errors were minimal and did not significantly affect the overall performance metrics.

Another perspective considered in this evaluation was the model’s behavior under different data distributions and streaming scenarios. Real-time testing was conducted to simulate a production environment where data is continuously arriving. The model was able to maintain its high-performance metrics without noticeable degradation, confirming that it can handle fast-paced traffic patterns and evolving attack strategies. This capability is essential for any intrusion detection system intended for live deployment, as static models often do not adapt to new or changing traffic characteristics. The results demonstrate that the proposed framework excels in several important dimensions: accuracy, fairness between classes, computational efficiency, real-time applicability, and robustness to older and newer attack scenarios. By consistently achieving high performance metrics and addressing common challenges such as data imbalance and real-time constraints, the framework establishes itself as a highly effective and practical solution for network intrusion detection.

### Interpretability of attack detection

To provide transparency into the model’s decision-making process, we incorporated post-hoc interpretability using SHAP SHapley Additive exPlanations (SHAP). SHAP values were computed for a subset of test predictions to quantify the contribution of each input feature to the model’s classification decisions. This analysis was applied to the refined embeddings generated by the Transformer-based autoencoder prior to final classification. Visualizations of feature contributions revealed that attributes such as num_root, srv_serror_rate, and dst_host_srv_count consistently had the highest SHAP impact in distinguishing attack traffic from benign flows.

The use of SHAP improved understanding of how the model separates different classes, particularly in multi-class scenarios involving rare attacks such as U2R and R2L. For instance, elevated values of num_shells and root_shell were shown to directly influence predictions toward the U2R class. These findings support the model’s practical value by offering explanations that align with known attack behavior signatures. By highlighting feature importance per prediction, the system can aid human analysts in verifying alarms, reducing uncertainty in automated decisions, and building operational trust in the deployed IDS. To explain how the model makes decisions, we employed SHAP (SHapley Additive exPlanations) to analyze the contribution of each input feature to classification outcomes. By computing SHAP values on test predictions, we identified key features such as num_root, srv_serror_rate, and dst_host_srv_count as strong indicators for attack classes. SHAP plots revealed how different feature values increased or decreased the likelihood of classifying a flow as malicious. These insights not only help validate the model’s internal logic but also enhance trust and transparency for security analysts reviewing alerts.

### Computational overhead and complexity analysis

The integration of s, Transformer-based autoencoders, and contrastive learning introduces additional computational layers compared to conventional intrusion detection models. To assess this impact, we evaluated the model’s parameter size, training.

time, inference time per sample, and memory usage. As shown in Table 7, the complete framework contains approximately

**Table 7:** Memory and computational efficiency.

Metric	Value
Model parameters	12.5 million
Inference memory usage	1.2 GB
Training time (1 RTX 3090)	24 h
Inference time Per Flow	2.3 ms

12.5 million parameters and maintains an inference memory footprint of 1.2 GB, which is manageable for deployment on standard GPU servers. Training time increases due to the sequential stages of graph embedding, attention-based encoding, and contrastive supervision. However, training is a one-time offline process, while inference remains efficient with an average processing time of 2.3 milliseconds per network flow. Despite the added components, the model’s runtime remained suitable for near real-time intrusion detection tasks. In terms of complexity, the GNN introduces *O*(*V* ^2^) operations during message passing, the Transformer introduces *O*(*n*^2^) self-attention cost, and contrastive loss requires pairwise similarity computations. These steps are parallelized during batch processing using modern deep learning libraries. Moreover, each component was lightweight in design: GCN layers used sparse adjacency matrices, the Transformer used 2 layers with 4 attention heads, and contrastive learning was applied only at the embedding stage. This modular architecture ensured that computational costs remained bounded while enabling a multi-perspective learning approach that boosted performance across all major metrics. The marginal increase in computational cost is justified by the observed gains in accuracy, interpretability, and robustness—especially in detecting minority-class and zero-day attacks. Therefore, the framework achieves a practical balance between performance and overhead, making it suitable for operational cloud or edge-based deployments.

### Justification of evaluation metrics

The evaluation metrics—accuracy, precision, recall, and F1-score—were chosen to capture both overall performance and the model’s behavior under class imbalance. While accuracy offers a broad view, it can be misleading when benign traffic dominates. Precision helps reduce false positives, minimizing alert fatigue, whereas recall is more critical in intrusion detection, where missing actual attacks poses greater risk than raising false alarms. The F1-score balances these concerns and is particularly useful for minority classes like U2R and R2L. By analyzing all four metrics, the evaluation provides a realistic and security-focused assessment of detection capability.

## Discussion and comparison

Compared to the studies described in Table 1, our proposed approach achieves consistently higher accuracy and precision, even under challenging conditions. For instance, while the Dugat-LSTM approach by Devendiran et al. achieves impressive results on TON-IOT and NSL-KDD, it suffers from high computational complexity. In contrast, our method maintains comparable accuracy levels without the need for excessive computational resources. Similarly, although the method of Talukder et al. to handle imbalanced data achieves high precision, it is highly dependent on oversampling, increasing processing time. Our model instead employs advanced loss functions and feature extraction techniques that effectively handle imbalances without inflating resource usage.

Furthermore, unlike the studies by Wang et al. and Manocchio et al., which focus on smaller or highly controlled datasets, our framework demonstrates superior generalization capabilities across multiple public datasets, including NSL-KDD and CIC-IDS-2018. This robustness shows our framework’s ability to handle various types of traffic patterns and attacks, a important requirement for real-world deployment.

A key differentiator lies in our method’s low false-positive rate. Although studies such as Tang et al. and Kim et al. have reported challenges with false positives, our approach consistently reduces these errors, resulting in a more reliable intrusion detection system. Additionally, our results confirm that even under multiclass classification scenarios, often a weak point for many models, our framework achieves high precision and recall across all categories.

**Fig. 7:**
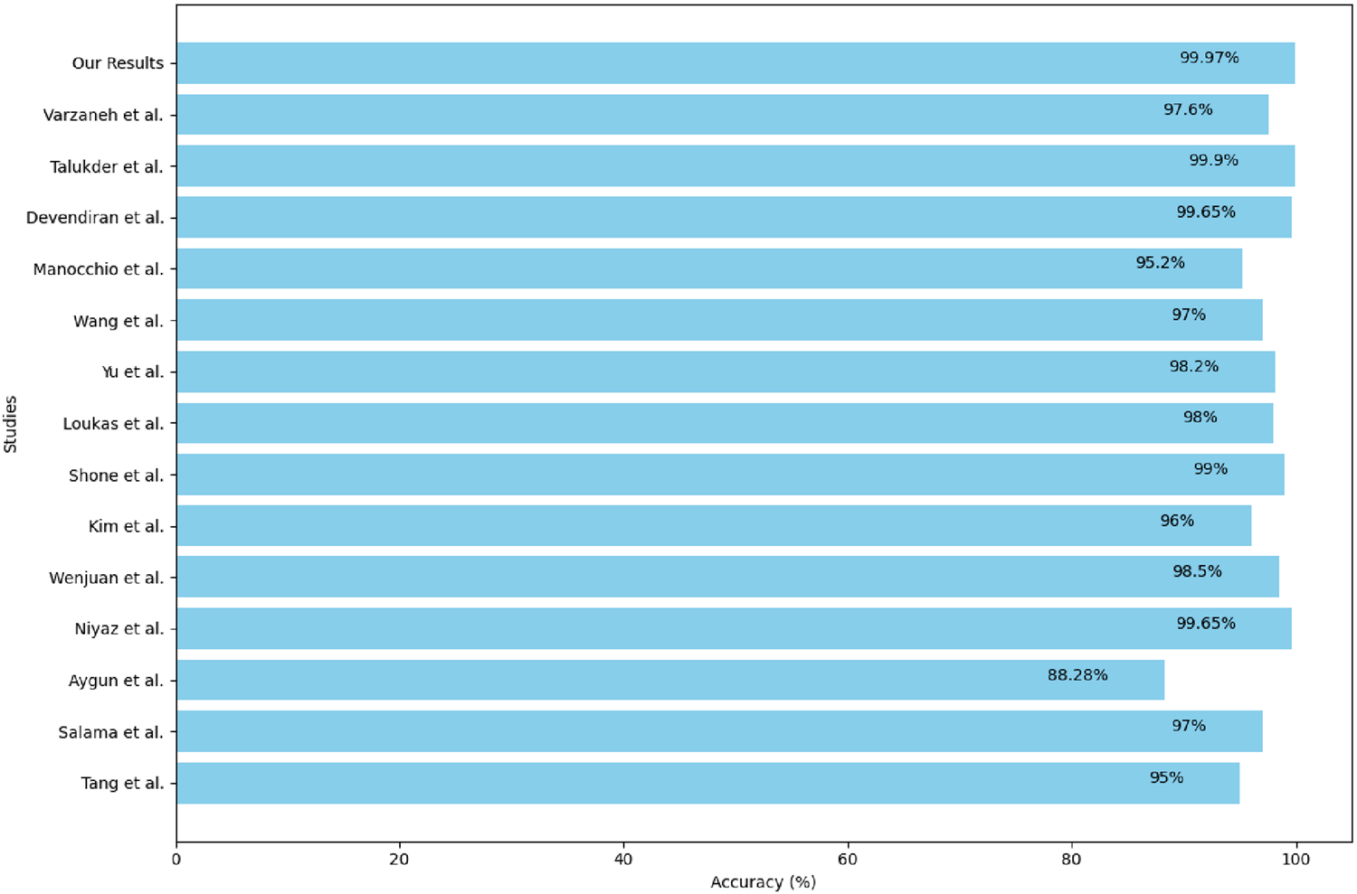
Comparison of accuracy and F1-scores between our approach and other state-of-the-art methods.

By consistently outperforming other approaches in terms of accuracy, computational efficiency, and robustness against class imbalance, our framework establishes a new benchmark for network intrusion detection. The inclusion of Figure 7 visually demonstrates these improvements, clearly showing how our model not only achieves state-of-the-art performance but also sets a higher standard for future research in this domain.

### Uniqueness of the proposed framework compared to related methods

The uniqueness of the proposed framework lies in its integrated use of graph-based embeddings, Transformer autoencoding, and contrastive learning within a unified pipeline. Traditional IDS models often rely on either feature-based deep neural networks or sequence-based LSTMs, which are limited in capturing relational dependencies and long-range interactions. In contrast, our use of allows the model to encode communication patterns across hosts and services, which is particularly valuable for identifying lateral movement and multi-hop attacks.

While some recent works have used Transformers for packet sequence modeling, they typically rely on flat, tokenized input and do not benefit from structured graph inputs. Our method uses a Transformer-based autoencoder to refine graph-derived embeddings, preserving both structural and contextual relationships. Additionally, most existing IDS models optimize only for classification loss. By integrating contrastive learning, our framework encourages better class separation in the latent space, leading to improved recall on underrepresented attack types. Compared to methods such as DBNs, CNNs, or sparse autoencoders that treat input independently, our architecture maintains end-to-end relational reasoning, temporal abstraction, and decision-space regularization. This multi-stage design is not only more robust to class imbalance but also more interpretable through post-hoc analysis like SHAP, making it practical for real-world threat response.

## Security analysis

### Formal security considerations

While this study focuses primarily on behavioral detection, formal validation of security properties in the underlying communication protocols and data flow remains important. Tools such as ProVerif and Scyther can be employed in future work to symbolically verify authentication, confidentiality, and integrity guarantees in communication between data sources and the IDS processing pipeline. In particular, the message exchange between traffic collectors, the graph constructor, and the Transformer inference module could be modeled using the Dolev-Yao intruder model. This would allow verification of properties such as replay resistance, tamper detection, and node impersonation resistance. At present, no protocol-level vulnerabilities are introduced by the inference pipeline; however, a formal security proof of confidentiality and correctness in the IDS communication flow has not been performed. Incorporating such symbolic analysis into the deployment pipeline is planned for future work to enhance protocol assurance.

### Informal threat model

The proposed intrusion detection framework assumes a trusted monitoring node that collects flow-level statistics and metadata from distributed network endpoints. Potential adversaries may attempt to evade detection by manipulating packet contents, mimicking benign traffic behavior, or launching coordinated multi-stage attacks (e.g., DDoS, U2R, R2L). The system is designed to detect such anomalies by analyzing structural patterns in traffic flows and embedding behaviors across hosts. The attack model assumes no control over the IDS infrastructure itself but permits adversaries to inject or replay malicious traffic. The model addresses attacks such as port scanning, privilege escalation, traffic flooding, and data exfiltration attempts. However, targeted poisoning of training data or adversarial perturbations at the model input level are not explicitly mitigated in this version and represent areas for future integration with robust learning.

## Conclusion

In this work, we have presented a novel Transformer-based framework for network intrusion detection that addresses key challenges such as class imbalance, real-time performance, and generalization to diverse attack types. By combining graph- based feature extraction, Transformer-based autoencoding, and contrastive learning, the proposed approach achieved strong performance across multiple benchmark datasets, including NSL-KDD and CIC-IDS-2018. With an average accuracy of 99.97% and low false-positive rates, the system demonstrates robust and reliable detection capabilities suitable for both conventional and cloud-based environments. In comparison to existing techniques, the proposed framework delivers improved accuracy and F1-scores while maintaining computational efficiency. Its modular architecture supports high-throughput inference, ensuring responsiveness in live network scenarios. Importantly, the model achieves balanced detection rates across both frequent and minority-class attacks, enhancing its applicability in environments where fair treatment of varied threat types is required. Despite these strengths, the approach has limitations in detecting subtle or persistent threats such as APTs, which often unfold over long durations using minimal behavioral signatures per session. Since the current model operates primarily at the flow level and relies on static labeled datasets, it may not capture the full temporal context or dynamic threat chains needed to identify stealthy multi-stage intrusions. Additionally, adversarial attacks and adaptive evasion strategies were not within the current evaluation scope. Future research will explore continual learning mechanisms, temporal graph modeling, and memory-augmented detection systems to address such evolving threats. Real-time adaptation, combined with formal security validation and deployment on distributed edge devices, also represents a promising direction to enhance operational utility. This research establishes a high-performance foundation while outlining the next steps toward more resilient, context-aware intrusion detection systems.

## Data Availability

The datasets generated and/or analyzed during the current study are available in the following repositories:—NSL-KDD dataset: https://www.kaggle.com/datasets/hassan06/nslkdd—CIC-IDS-2018 dataset: https://www.unb.ca/cic/datasets/ids-2018.html.

## References

[1] Obi, O. C. et al. Review of evolving cloud computing paradigms: Security, efficiency, and innovations. *Comput. Sci. & IT Res. J.* **5**, 270–292 (2024).

[2] Sharif, F. The role of ensemble learning in strengthening intrusion detection systems: A machine learning perspective. *Int. J. Comput. Eng. Technol.* (2024).

[3] Sharma, H. The evolution of cybersecurity challenges and mitigation strategies in cloud computing systems. *Int. J. Comput. Eng. Technol.* **15**, 118–127 (2024).

[4] Kandhro, I. A. et al. Detection of real-time malicious intrusions and attacks in iot empowered cybersecurity infrastructures. *IEEE Access* **11**, 9136–9148 (2023).

[5] Chukwunweike, J. N., Adewale, A. & Osamuyi, O. Advanced modelling and recurrent analysis in network security: Scrutiny of data and fault resolution. *World J. Adv. Res. Rev.* **23**, 2373–2390 (2024).

[6] Sengupta, S. et al. A review of deep learning with special emphasis on architectures, applications and recent trends. *Knowl. Based Syst.* **194**, 105596 (2020).

[7] Zhang, C., Wang, N., Hou, Y. T. & Lou, W. Machine learning-based intrusion detection systems: Capabilities, methodolo- gies, and open research challenges. *Authorea Prepr.*

[8] Attou, H. et al. Towards an intelligent intrusion detection system to detect malicious activities in cloud computing. *Appl. Sci.* **13**, 9588 (2023).

[9] Tabrizchi, H. & Kuchaki Rafsanjani, M. A survey on security challenges in cloud computing: issues, threats, and solutions. *J. supercomput.* **76**, 9493–9532 (2020).

[10] Shamshirband, S. et al. Computational intelligence intrusion detection techniques in mobile cloud computing environments: Review, taxonomy, and open research issues. *J. Inf. Secur. Appl.* **55**, 102582 (2020).

[11] Ennaji, S., De Gaspari, F., Hitaj, D., Kbidi, A. & Mancini, L. V. Adversarial challenges in network intrusion detection systems: Research insights and future prospects. arXiv preprint arXiv:2409.18736 (2024).

[12] Ahmed, U. et al. Signature-based intrusion detection using machine learning and deep learning approaches empowered with fuzzy clustering. *Sci. Reports* **15**, 1726 (2025). 10.1038/s41598-025-85866-7 PMC11724853 39799225

[13] Buchta, R., Gkoktsis, G., Heine, F. & Kleiner, C. Advanced persistent threat attack detection systems: A review of approaches, challenges, and trends. *Digit. Threat. Res. Pract.* **5**, 1–37 (2024).

[14] Kocher, G. & Kumar, G. Machine learning and deep learning methods for intrusion detection systems: recent developments and challenges. *Soft Comput.* **25**, 9731–9763 (2021).

[15] Qaddos, A. et al. A novel intrusion detection framework for optimizing iot security. *Sci. Rep.* **14**, 21789 (2024). 39294195 10.1038/s41598-024-72049-z PMC11410947

[16] Tang, T. A., Mhamdi, L., McLernon, D., Zaidi, S. A. R. & Ghogho, M. Deep learning approach for network intrusion de- tection in software defined networking. In *2016 international conference on wireless networks and mobile communications (WINCOM)*, 258–263 (IEEE, 2016).

[17] Salama, M. A., Eid, H. F., Ramadan, R. A., Darwish, A. & Hassanien, A. E. Hybrid intelligent intrusion detection scheme. In *Soft computing in industrial applications*, 293–303 (Springer, 2011).

[18] Aygun, R. C. & Yavuz, A. G. Network anomaly detection with stochastically improved autoencoder based models. In *2017 IEEE 4th international conference on cyber security and cloud computing (CSCloud)*, 193–198 (IEEE, 2017).

[19] Javaid, A., Niyaz, Q., Sun, W. & Alam, M. A deep learning approach for network intrusion detection system. In *Proceedings of the 9th EAI International Conference on Bio-inspired Information and Communications Technologies (formerly BIONETICS)*, 21–26 (2016).

[20] Niyaz, Q., Sun, W. & Javaid, A. Y. A deep learning based ddos detection system in software-defined networking (sdn). arXiv preprint arXiv:1611.07400 (2016).

[21] Wang, W., Du, X., Shan, D., Qin, R. & Wang, N. Cloud intrusion detection method based on stacked contractive auto-encoder and support vector machine. *IEEE Trans. Cloud Comput.* **10**, 1634–1646 (2020).

[22] Kim, J., Shin, N., Jo, S. Y. & Kim, S. H. Method of intrusion detection using deep neural network. In *2017 IEEE international conference on big data and smart computing (BigComp)*, 313–316 (IEEE, 2017).

[23] Papamartzivanos, D., Mármol, F. G. & Kambourakis, G. Introducing deep learning self-adaptive misuse network intrusion detection systems. *IEEE access* **7**, 13546–13560 (2019).

[24] Shone, N., Ngoc, T. N., Phai, V. D. & Shi, Q. A deep learning approach to network intrusion detection. *IEEE Trans. Emerg. Top. Comput. Intel.* **2**, 41–50 (2018).

[25] Loukas, G. et al. Cloud-based cyber-physical intrusion detection for vehicles using deep learning. *Ieee Access* **6**, 3491–3508 (2017).

[26] Yu, Y., Long, J. & Cai, Z. Network intrusion detection through stacking dilated convolutional autoencoders. *Secur. Commun. Networks* **2017**, 4184196 (2017).

[27] Abdulganiyu, O. H., Tchakoucht, T. A. & Saheed, Y. K. Towards an efficient model for network intrusion detection system (ids): Systematic literature review. *Wirel. Netw.* **30**, 453–482 (2024).

[28] Wang, X. et al. Advanced network intrusion detection with tabtransformer. *J. Theory Pract. Eng. Sci.* **4**, 191–198 (2024).

[29] Manocchio, L. D. et al. Flowtransformer: A transformer framework for flow-based network intrusion detection systems. *Expert. Syst. with Appl.* **241**, 122564 (2024).

[30] Devendiran, R. & Turukmane, A. V. Dugat-lstm: Deep learning based network intrusion detection system using chaotic optimization strategy. *Expert. Syst. with Appl.* **245**, 123027 (2024).

[31] Talukder, M. A. et al. Machine learning-based network intrusion detection for big and imbalanced data using oversampling, stacking feature embedding and feature extraction. *J. big data* **11**, 33 (2024).

[32] Varzaneh, Z. A. & Hosseini, S. An improved equilibrium optimization algorithm for feature selection problem in network intrusion detection. *Sci. Rep.* **14**, 18696 (2024). 39134565 10.1038/s41598-024-67488-7 PMC11319621

[33] Shitharth, S., Mohammed, G. B., Ramasamy, J. & Srivel, R. Intelligent intrusion detection algorithm based on multi-attack for edge-assisted internet of things. In *Security and risk analysis for intelligent edge computing*, 119–135 (Springer, 2023).

[34] Selvarajan, S., Shaik, M., Ameerjohn, S. & Kannan, S. Mining of intrusion attack in scada network using clustering and genetically seeded flora-based optimal classification algorithm. *IET Inf. Secur.* **14**, 1–11 (2020).

[35] Prashanth, S., Shitharth, S., Praveen Kumar, B., Subedha, V. & Sangeetha, K. Optimal feature selection based on evolutionary algorithm for intrusion detection. *SN Comput. Sci.* **3**, 439 (2022).

[36] Rabie, O. B. J. et al. A novel iot intrusion detection framework using decisive red fox optimization and descriptive back propagated radial basis function models. *Sci. Rep.* **14**, 386 (2024). 38172185 10.1038/s41598-024-51154-z PMC10764843

[37] Shitharth, S., Kshirsagar, P. R., Balachandran, P. K., Alyoubi, K. H. & Khadidos, A. O. An innovative perceptual pigeon galvanized optimization (ppgo) based likelihood naïve bayes (lnb) classification approach for network intrusion detection system. *IEEE Access* **10**, 46424–46441 (2022).

[38] Tellache, A., Mokhtari, A., Korba, A. A. & Ghamri-Doudane, Y. Multi-agent reinforcement learning-based network intrusion detection system. In *NOMS 2024–2024 IEEE Network Operations and Management Symposium*, 1–9 (IEEE, 2024).

[39] Korba, A. A., Boualouache, A. & Ghamri-Doudane, Y. Zero-x: A blockchain-enabled open-set federated learning framework for zero-day attack detection in iov. *IEEE Trans. Veh. Technol.* **73**, 12399–12414 (2024).

[40] Diaf, A., Korba, A. A., Karabadji, N. E. & Ghamri-Doudane, Y. Bartpredict: Empowering iot security with llm-driven cyber threat prediction. In *GLOBECOM 2024–2024 IEEE Global Communications Conference*, 1239–1244 (IEEE, 2024).

[41] Hyatt, J.-P. K., Bienenstock, E. J. & Tilan, J. U. A student guide to proofreading and writing in science. *Adv. Physiol. Educ.* (2017). 10.1152/advan.00004.2017 28679566

[42] Diaf, A., Ghamri-Doudane, Y. *et al.* Ai-driven fast and early detection of iot botnet threats: A comprehensive network traffic analysis approach. arXiv preprint arXiv:2407.15688 (2024).

[43] Karabadji, N. E., Ghamri-Doudane, Y. *et al.* Zero-day botnet attack detection in iov: A modular approach using isolation forests and particle swarm optimization. arXiv preprint arXiv:2504.18814 (2025).

[44] Korba, A. A., Diaf, A. & Ghamri-Doudane, Y. Ai-driven fast and early detection of iot botnet threats: A comprehensive network traffic analysis approach. In *2024 International Wireless Communications and Mobile Computing (IWCMC)*, 1779–1784 (IEEE, 2024).

[45] Manda, V. K., Christy, V. & Hlali, A. Current trends, opportunities, and futures research directions in geospatial technologies for smart cities. *Recent Trends Geospatial AI* 239–270 (2025).

[46] Zhou, G. & Barbieri, S. Generating clinically realistic ehr data via a hierarchy-and semantics-guided transformer. arXiv preprint arXiv:2502.20719 (2025).

[47] Kheddar, H. Transformers and large language models for efficient intrusion detection systems: A comprehensive survey. arXiv preprint arXiv:2408.07583 (2024).

[48] Yue, Y., Chen, X., Han, Z., Zeng, X. & Zhu, Y. Contrastive learning enhanced intrusion detection. *IEEE Trans. Netw. Serv. Manag.* **19**, 4232–4247 (2022).

[49] Abdulganiyu, O. H., Tchakoucht, T. A., Saheed, Y. K. & Ahmed, H. A. Xidintfl-vae: Xgboost-based intrusion detection of imbalance network traffic via class-wise focal loss variational autoencoder. *The J. Supercomput.* **81**, 1–38 (2025).

